# Changes in sleep EEG with aging in humans and rodents

**DOI:** 10.1007/s00424-021-02545-y

**Published:** 2021-04-01

**Authors:** Diana Campos-Beltrán, Lisa Marshall

**Affiliations:** 1 Institute of Experimental and Clinical Pharmacology and Toxicology, University of Lübeck, Ratzeburger Allee 160, 23562 Lübeck, Germany; 2 Center for Brain, Behavior and Metabolism, University of Lübeck, Ratzeburger Allee 160, 23562 Lübeck, Germany

**Keywords:** Aging, Sleep, Electrophysiology, EEG, Human, Rodents

## Abstract

Sleep is one of the most ubiquitous but also complex animal behaviors. It is regulated at the global, systems level scale by circadian and homeostatic processes. Across the 24-h day, distribution of sleep/wake activity differs between species, with global sleep states characterized by defined patterns of brain electric activity and electromyography. Sleep patterns have been most intensely investigated in mammalian species. The present review begins with a brief overview on current understandings on the regulation of sleep, and its interaction with aging. An overview on age-related variations in the sleep states and associated electrophysiology and oscillatory events in humans as well as in the most common laboratory rodents follows. We present findings observed in different studies and meta-analyses, indicating links to putative physiological changes in the aged brain. Concepts requiring a more integrative view on the role of circadian and homeostatic sleep regulatory mechanisms to explain aging in sleep are emerging.

Both aging and sleep affect as well as depend upon complex mechanisms involving processes at different structural levels. At the global level, the ventrolateral preoptic nucleus located in the hypothalamus has long been considered the sleep center. However, the existence of wake-sleep regulatory circuits within the hypothalamus, brainstem, and basal forebrain, characterized by functionally specific cell types, has since been disclosed. Findings show that no one nucleus contains only, e.g., sleep-active neurons, but that functionally specific mostly GABA and glutamatergic cell types are co-distributed with varying predominance in different regions or nuclei [46]. The discharge of these cells is regulated by neuromodulators such as acetylcholine, orexin, or norepinephrine, which vary in their action and efficacy. For instance, non-synaptic release of norepinephrine from neurons of the locus coeruleus (LC) enables spatially widespread efficacy, while, at the same time, LC neuronal activity occurs phase-locked to the sleep slow oscillation [86]. In the case of orexin, activity is key for maintained wakefulness, as indicated by the characteristic wakefulness deficit in narcolepsy when hypothalamic orexinergic activity is impaired [47]. Ascending fibers innervate the neocortex and hippocampus which reflect changes in behavioral state and neuronal activity as well as age-dependent modifications [e.g., 59, 77]. Due to their laminar cytoarchitecture, the neocortex and hippocampus can generate far field potentials, and their electrophysiological signals, especially brain rhythms of the electroencephalogram (EEG), are readily used to assess sleep states in mammals.

In general, large-scale age-related structural changes such as cortical thinning, white matter degeneration, neurotransmitter dysregulation, and/or receptor distribution affect sleep and its electrophysiological representation [27, 78, 120]. Age-related alterations of many structures and basic physiological mechanisms addressed in this volume likely also affect sleep and sleep/wake processes, e.g., astroglial aging (Verkhratsky & Semyanov, this volume) and changes in energy metabolism (Lushchak, this volume) as well as in vascular and hemodynamic properties (Robinson, this volume). Furthermore, we refer here to some comprehensive recent reviews on sleep-associated age-related changes in the transcriptome and on epigenetic aging [2, 38, 39, 98].

Sleep is endowed with a multitude of functions, not only neuronal, e.g., memory consolidation or synaptic downscaling, but also at immune and metabolic levels [4, 53, 63, 99, 107]. In fact, strong evidence suggests that brain rhythms during sleep are more than merely correlates of neuronal activity. In particular, the slow oscillations of non-rapid eye movement sleep (NREMS) are suggested to support immune function [5] and serve waste clearance by reinforcing the glymphatic system function [3]. More recently, studies lend evidence to a restorative function of sleep for the genome, e.g., for DNA break down repair [69].

We aim here to highlight the most robust findings on the impact of aging on sleep in three mammalian species (Fig. 1) as assessed by brain electric activity. The first section gives an overview on the regulation of sleep and the second to fourth on age-related effects on sleep EEG in humans, laboratory mice, and rats respectively.

**Fig. 1:**
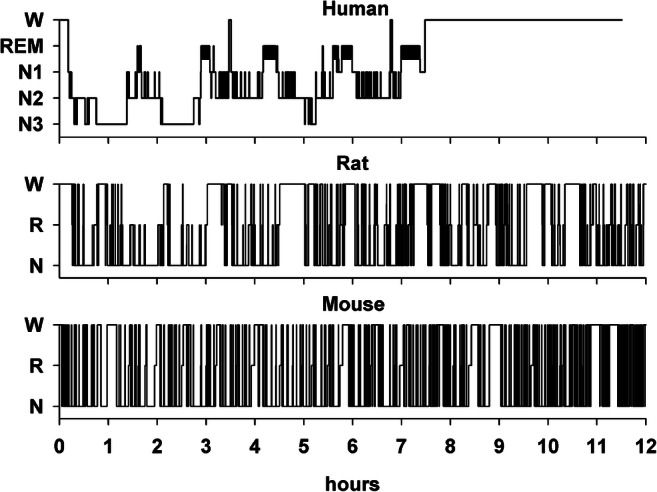
Comparison of the normal sleep architecture during the inactive sleep period of the three species discussed in this review. Lights out was at 0 h for the human subject and sleep was recorded during 8 h. The rodent species were held on a 12:12 L:D regime, with lights on depicted at 0 h and sleep recorded through 12 h. Sleep architecture is determined from EEG and electromyography recordings. Note the typical difference in sleep cycle lengths across species. W wake, REM rapid eye movement sleep, stages N1–N3 in human sleep; W wake, R rapid eye movement sleep, N non-rapid eye movement sleep in the rodent species. Rat data were kindly provided by Gina R. Poe, University of California, Los Angeles (UCLA)

## Regulation of sleep

How is sleep regulated? The most widely used conceptual model for sleep regulation for the timing and intensity of sleep at the systems level is the Two Process Model by Borbély, first published in 1982 and extensively updated in 2016 [9]. At its core, sleep is regulated by the interaction of a process controlled by the circadian pacemaker C (Process C) and a homeostatic process S (Process S). Thus, effects of aging on functions within either of these two processes may affect sleep. Relevant EEG markers for Process S are slow wave activity (SWA), and theta activity. Both frequency bands reveal regional differentiation, putatively linked to plasticity-related processes of learning during wakefulness and synaptic homeostasis during sleep [9, 44]. The homeostatic increases in SWA in response to prolonged prior wakefulness are blunted in older relative to younger subjects in humans [55, 61, 70] and rodents [17, 51, 65, 82, 101]. Features of slow waves are discussed below.

The major substance ascribed a homeostatic, centrally mediated function in Process S and expressed in magnitude of EEG slow wave activity is adenosine. Neuronal activity during waking leads via neuro-glial circuitry to a global increment in extracellular adenosine concentrations, with a decrease adenosine occurring during the rest phase [6, 9]. Aging is suggested to impact homeostatic function in cortical and subcortical regions by modulating production of adenosine, reduced binding sites, binding potential, or efficacy [15, 27, 68, 71, 78]. Recently, using optogenetic activation, glutamatergic neurons in the basal forebrain were found critically involved in this sleep homeostatic dynamics of adenosine [84]. Mainly protein levels of vesicular glutamate transporter 2 (vGluT2), which transports glutamate into secretory vesicles, reveal age-related changes [94]. Together with the finding that sleep/wake activity drives posttranscriptional processes in forebrain synaptosomes [75], an age-dependent shift in transcriptional vs. posttranscriptional activity may contribute to aging sleep [48].

Which processes are associated to Process C? Melatonin and core body temperature represent the dominant markers of the central pacemaker, the suprachiasmatic nucleus (SCN). The melatonin nocturnal level is strongly reduced from young to middle-aged/elderly [109, 121]. The circadian core body temperature rhythm is also weakened in aging [111]. Both the circadian phase of melatonin and of core body temperature are reported to move earlier, i.e., phase advance, with age as compared to young adults, yet age-related changes in sleep timing are not attributed to a shortening of circadian period [25]. Common to humans and most rodents, a decrease in amplitude or functional impairment in circadian rhythm is observed, which some studies indicate result from age-related changes in the molecular machinery of the SCN and non-SCN clocks [18, 23, 73, 114, 119].

A study by Cajochen and colleagues [10] compared age-related changes in the circadian and homeostatic regulation of sleep between elderly and young adults and concluded that weaker circadian regulation rather than homeostatic regulation underlies age-related changes in sleep. They measured significantly reduced melatonin secretion, reduced circadian modulation of rapid eye movement sleep (REMS), and sleep spindle frequency, but homeostatic responses (measured as SWA response to different levels of sleep deprivation) were only selectively reduced, i.e., over the frontal cortex. This relatively stronger effect of age-related changes in Process C than in Process S is reflected in other studies on circadian-sleep interaction. Sleep in older as compared to younger subjects is much more vulnerable to circadian misalignment [25], which might be due to a reduced sensitivity or responsiveness of the suprachiasmatic nucleus (SCN) to environmental cues and non-image forming functions to light [20, 26].

One major hub for the interaction of aging circadian and homeostatic functions is the hypothalamus and related systems [49, 90]. For instance, melatonin directly inhibits several responses to the adrenocorticotropic hormone (ACTH) in the human adrenal gland, such as cortisol and progesterone production [11]. Cortisol is the peripheral end-point of the neuroendocrine hypothalamic-pituitary-adrenocortical system that interacts reciprocally with the hypothalamic-pituitary-somatotrophic system, thus suggesting convergent age-dependent regulators of sleep-related hormone secretion and sleep EEG [40, 105, 108]. Thus, any of the above structures or functions that change over the course of a lifespan can thereby clearly impact sleep. In the original Two Process Model, Processes C and S were regulated independently and represented global entities only. Advances at the biochemical and molecular level have deepened our understanding on the complexity of sleep regulatory processes and disclosed common signaling pathways for regulation of the two global processes [52]. Non-SCN circadian clocks in the brain and in the periphery affect sleep [31, 34, 66, 90]. Moreover, extra-SCN clock gene expression in the brain, especially in the cortex, is dependent upon prior sleep-wake activity [9, 19]. In fact, Noya and colleagues revealed that gene expression in synapses is regulated in a circadian fashion while gene translation occurs in response to sleep/wake activity [75].

Furthermore, the concept of an essential contribution of local to global sleep has arisen [52]. Most intensely investigated (local) sleep regulatory substances, i.e., substances that are synthetized or released in an activity-dependent way, and involved in homeostatic regulation are proinflammatory cytokines, such as tumor necrosis factor alpha and interleukin 1b, or nitric oxide. These substances and/or their signaling cascades reveal age-dependent changes [29, 37, 53, 54].

## Age-related effects on sleep EEG in humans

We define healthy aging human subjects in accordance to a review by Scullin [100] as ≥ 60 years old, with young and middle-aged defined as < 30 years and 30–60 years old, respectively. A meta-analyses by Ohayon and colleagues [76] including way over 20 studies used slightly different borders, and included in addition a group of old elderly (≥ 70 years). Of all polysomnographic features, only sleep efficiency, i.e., the ratio of total sleep time (TST) compared to the total time in bed, was lower in this group, attributed most likely to increased sleep fragmentation: From 30 to 60 years of age, wake after sleep onset increased strongly, by about 10 min per decade, and revealed a strong effect size in the meta-analyses of Ohayon and colleagues. However, after the age of 60, neither TST, times spent in the different sleep stages, nor wake time after sleep onset changed significantly.

Martin and colleagues reported a moderate increase in sleep latency, i.e., the time required after lights out to reach sleep, with age [64]. Increases seem most apparent within the years after the 30s [58, 76]. Overall, from young to elderly, the amount of time spent in slow wave sleep (SWS) and REMS decreases, whereas time in lighter NREMS stages N2 and N1 increases [13, 58, 64, 76]. Interestingly, although effects of aging within males and females were generally similar, in the meta-analyses by Ohayon and colleagues [76], larger effect sizes were observed for women in TST, sleep efficiency, and percentage in stage 1 sleep, meaning that age effected these parameters in women more strongly than in men. Women revealed longer TST but also longer sleep latency, than similarly aged men. However, they revealed a greater percentage of SWS as well as less percentage of stage 2 sleep. Despite differences in magnitude in some parameters, the direction of change in both genders was similar. It appears gender differences more than an interaction between gender and age exist [36]. Not considered in this review are the interactions of sleep with menstrual cycle and sex-related circadian variations [8]. A lower percentage of time spent in REMS is frequently reported in middle-aged compared to young subjects, but no further decrease in elderly [60, 62, 64].

It is to note that not all studies comparing nocturnal sleep in elderly with that of younger adults mention in the methods whether daytime napping occurred. As discussed intensely by Li et al. [58]. this is no trivial matter, since napping effects Process S. Napping is more frequent in older subjects, yet omission of habitual napping for experimental purposes may bias results, if we are interested in measuring “normal sleep behavior” within a 24-h period. Obviously, permitting habitual naps influences nocturnal sleep drive, and the ability to match parameters of napping such as time of day and duration presents a further challenge. Cognitive performance and physiological (e.g., cardiovascular) functions have been associated positively with napping, thus potentially introducing a further bias when including or excluding habitual nappers [30, 67, 74]. In addition to changes in spontaneous sleep, aging may involve a decreased capacity to respond or decreased sensitivity to the accumulation of wake-associated substances. Several studies report that nocturnal sleep deprivation is associated with less SWS rebound in middle-aged than young adults [36, 93].

**Fig. 2:**
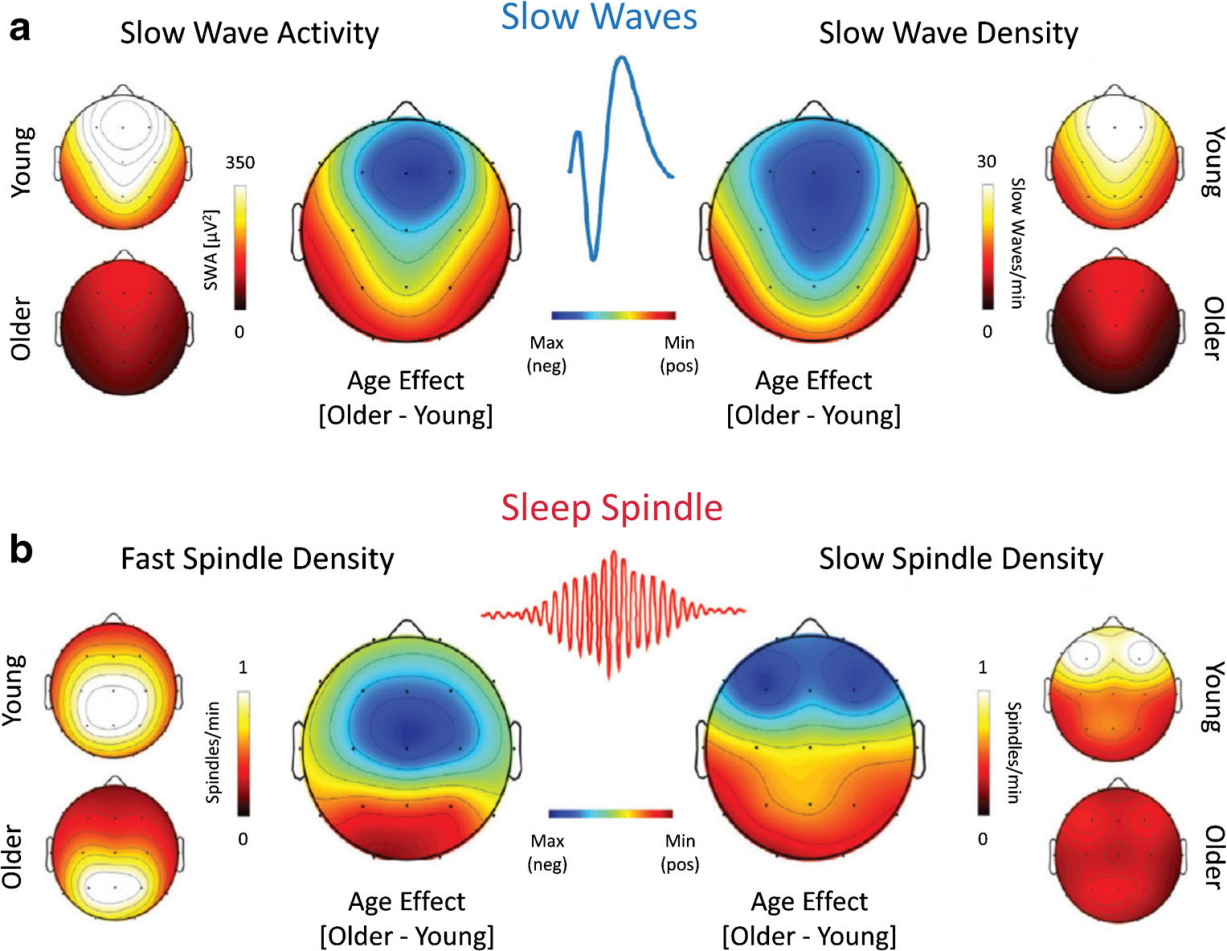
Age-related modulations in slow wave activity and sleep spindle density in humans predominate over frontal cortical regions. **a** Representative head plots of slow wave activity (< 4.6 Hz, absolute power as measured in μV^2^; left) and density (number of SW/min; right); warmer colors indicate higher values. The middle topoplots reveal the topographical differences in EEG activity between young and older human adults in which the cooler colors indicate greater differences. **b** Differences in the aged EEG activity in the fast sleep spindle density (13.5–15 Hz; bottom left) and slow sleep spindle density (12–13.5 Hz; bottom right). Figure taken and modified with permission from Mander and colleagues, Neuron (2017) 94 (1):19–36 [61]

Information provided from sleep stages and the amplitude or power of EEG brain rhythms is often complimentary. Consistent with reductions in time spent in SWS, spectral power of slow wave activity (SWA, < 4 Hz) is reduced in middle-aged and further in older compared to young adults (reviewed in [61]). The slow wave/slow oscillation features amplitude and density largely decrease with aging [13, 24, 61, cp. Fig. 2]. Decreased SWA and thus glymphatic system function may potentially lead to increased accumulation of toxic brain waste. Notably, several lifestyle factors aside from sleep also influence the glymphatic system [88].

In the course of the night, slow wave slope typically becomes less steep with decreased homeostatic drive [89]. In middle-aged as compared to younger subjects, nocturnal slow wave slopes are decreased, even after controlling for the effect of slow wave amplitude [13]. Absolute theta power was likewise reported to decline with age in nocturnal sleep within the first sleep cycles [36]. Studies imply that slow wave and theta activity share cortical network properties [32, 50].

Whereas N2 duration characteristically increases with age, after reaching a maximum density, duration, and length of sleep, spindles decrease, however with different dynamics. Whereas spindle density declines steadily from adolescence onward to old age, spindle amplitude peaks during childhood and decreases steadily with age. Spindle length peaks already early in life. From young adults to elderly, spindle topography shifts from a wider to a narrower distribution restricted to central sites [16]. In general, the decrement from young to middle-aged adults in spindle power, spindle density and amplitude, slow wave power, amplitude, density, and slope occurs predominantly over frontal/prefrontal regions (Fig. 2) [61, 64, 104]. Impaired spindle features have been speculated to be associated with both reduced white and gray matter and thalamocortical circuit changes (Fig. 2) [16, 61, 62]. In fact, selective atrophy within the medial frontal cortex in older adults predicted a lower degree of SW-spindle coupling of subjects [42].

## Chronobiological age and studies in rodents

Rodents are nocturnal animals and sleep mostly, but not only, during the day in short periods with frequent short awakenings [103, 113, c.p. Fig. 1], thus studies on sleep usually investigate the whole 24-h light-dark phase. In laboratory animals, typically a 12:12 light regime, consisting of 12-h light (inactive) phase and 12-h dark (active) phase, is maintained. The lifespans of mice with about 24 months [45] and of rats with about 3 years [87] are significantly shorter than of humans. Adult mice are mature at an age of 3–6 months, which corresponds to young adults between 20 and 30 years. Mice of 10–14 months are considered middle-aged corresponding to humans between 38 and 47 years and old mice range from 18 to 24 months corresponding to 56–69 years in humans [33]. Adult rats are mature at 6–12 months, middle-aged at about 18 months, and old at an age above 24 months [1]. However, as in human studies, defined age ranges differ between research groups.

Methods utilized for measuring the electrophysiological activity during sleep in laboratory rodents differ from typical human measurements. Human scalp electrodes assess the activity of larger cortical networks as compared to the invasive EEG electrodes in rodents. Local field potential recordings reflect activity of even smaller networks [21, 65].

## Age-related effects on sleep electrophysiology in mice

Most investigations on aging were conducted with the C57BL strain [28, 65, 72, 82, 102, 103, 115, 117] while a few studies investigated other strains, e.g., DBA/2J [28], the wild type from Tg2576 [43], or CBA/J [83]. Age-related changes in sleep EEG have been observed (see below), albeit they differ slightly among strains [28, 41]. Furthermore, almost all studies except for one [43] investigated male mice. Sleep in both sexes was analyzed and compared only once by Sigalas and colleagues [102]. In general, measured sleep parameters across research groups are less consistent than in human research; thus, degree of generalization is lower.

**Fig. 3:**
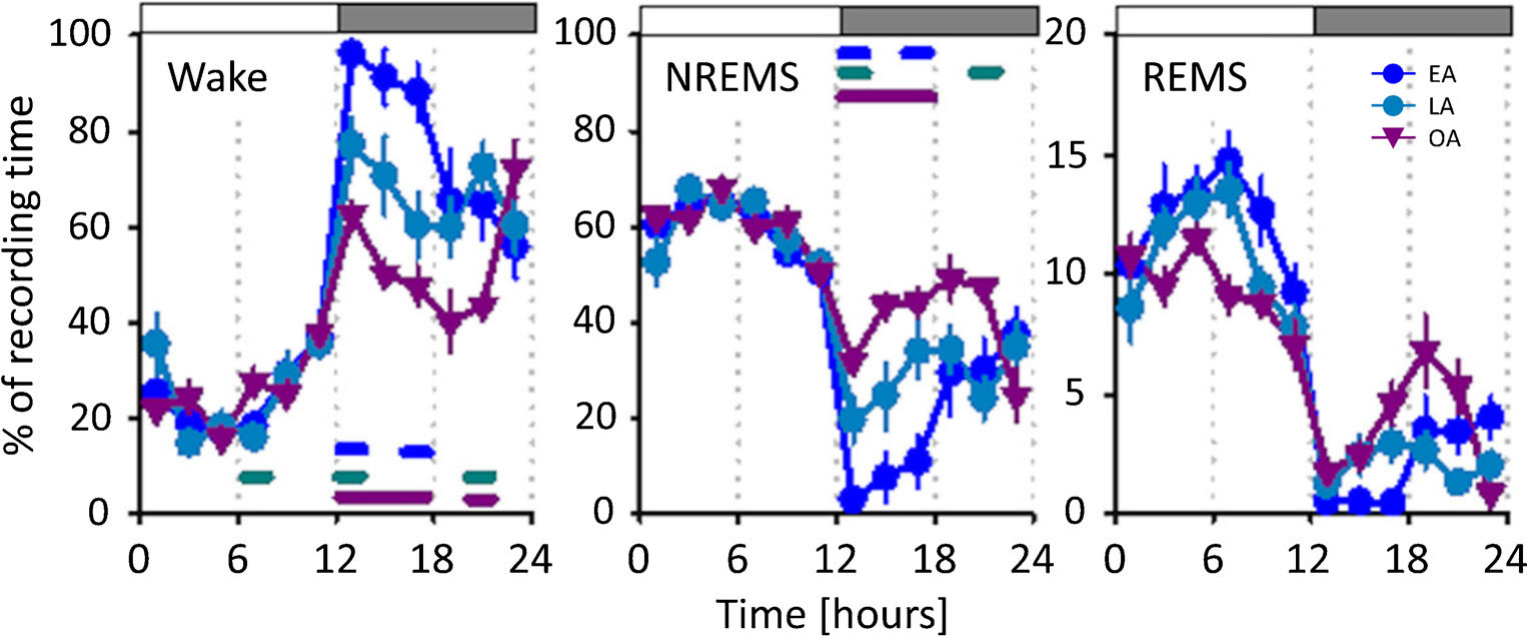
Age-related differences of sleep states within a sleep-wake cycle of undisturbed sleep in C57BL/6J mice. Diagrams show the time course of wake (left), NREMS (central), and REMS (right) in percentage (mean ± SEM) across a 24-h recording period. White and gray top bars indicate the light (inactive) and dark (active) phases, correspondingly. Comparisons are made between early adults (EA, blue), late adulthood (LA, cyan), and old adults (OA, purple) with significant differences comparing EA vs LA (blue), LA vs OA (cyan), and EA vs OA (purple). Data indicate that old mice had a significantly decreased wake and increased NREMS time as compared to younger animals, an effect that is more evident during their active phase. Figure taken and modified with permission from McKillop and colleagues, J Neurosci (2018) 38(16):3911–3928 [65]

The most consistent result among studies is an increase in NREMS at old ages across a 24-h period [65, 103, 115, 117]. This increment in NREMS corresponds mostly to the augmentation of NREMS during the dark phase [82, 103, 115, 117]. Decreased time spent awake, again especially during the dark phase, appears characteristic of aged mice [65, 82, 103, 115, 117]. TST across a 24-h period measured by electrophysiology [65] and by piezoelectric tracking of activity by Paulose et al. [83] also increased with age. Overall, there were no significant changes in REMS time with age in mice (Fig. 3) [65, 103, 117].

Similar to humans, mice present more fragmentation in their sleep during healthy aging. When analyzing sleep and wake bouts (bout refers to the consecutive epochs of a given stage), most studies reported difficulties in wake maintenance and higher sleep pressure that is more evidenced during the dark phase [72, 82, 115, 117]. Whereas SWA in the dark phase is often increased in aged mice [65, 82], Wimmer et al. measured a decrease in the relative SWA during the dark phase [117]. An increase in sleep pressure introduced by sleep deprivation, produced in older animals a lower rebound in SWA or a reduced decay rate, suggested to reflect a reduced capacity toward a homeostatic recovery process [41, 65, 82]. To specifically investigate homeostatic features, Panagiotou and colleagues measured slow oscillation/slow wave parameters, and found an increased amplitude and steeper amplitudes underscoring the concept that healthy older mice live under a condition of higher sleep pressure [82]. Theta activity during wakefulness is a correlate of arousal and is reported to positively correlate SWA [112]. Theta peak frequency during REMS in the light phase as well as during wake in the dark phase was slower in older mice suggesting reduced vigilance [117], but no other significant differences were observed for REMS [82, 117].

As detailed by McKillop and colleagues, differences in sleep with aging between humans and mice are associated with their discrepant ecological roles, brain/body size, and metabolism [65]. At a finer scale, not only different strains but substrains of wild-type mice reveal significantly different behavior which may well impact sleep and the expression of aging [12, 35]. As in humans, sleep EEG in old mice may be modulated by external variables such as exercise [81], diet [79], and light regime [80].

Two of the studies investigating age-related effects on sleep EEG in mice performed their experiments in vitro using brain slices. Sigalas et al. [102] analyzed the barrel cortex in slices with a thickness of 400 μm and found a shortened duration of the UP states in the old group, but no differences in either UP states’ density, slow oscillation peak amplitude, peak latency, or in the relative power of the frequencies within the delta to gamma bands. On the other hand, in stratum pyramidale of the CA1 area in hippocampal slices (450 μm thickness), Hermann et al. [43] found a decline in both the sharp wave frequency and the ripple oscillation energy with age that can be explained by a loss in synaptic strength and presynaptic plasticity in the area.

Naidoo and colleagues found a failure of wake orexinergic and noradrenergic neurons in aged mice to increase activity (as measured by c-fos) in response to sleep deprivation, with activity significantly lower than in young mice. This decreased responsiveness of wake active neurons may contribute to wake instability (e.g., shorter duration of wake bouts in the active phase) and dysregulation in the wake/sleep rhythm in aged mice [72]. Orexin (hypocretin) knockout mice, which serve as a model for narcolepsy [14], a sleep disorder in which a loss of orexin has been observed in humans [85, 106], show an increment in sleep intrusion episodes and hypersomnolence during the dark phase. A lower sensitivity to sleep pressure in aging is supported by findings of reduced adenosine A_1_ receptor levels in the hippocampus, cortex, basal ganglia, thalamus, and cerebellum of old mice [27, 78].

## Age-related effects on sleep electrophysiology in rats

The effect of healthy aging in the sleep electrophysiology has been investigated in different strains, such as Fisher 344 rats [51, 71, 92, 101, 116], Sprague-Dawley [101, 118], Wistar [17, 110], and Long Evans [57, 96]. All studies were performed in males, except the study by Kostin et al. [51] that used and compared both sexes. Since strain differences in rat sleep have been discovered [91, 101], the age-related effects will be described by strain.

Results on aging in Fisher 344 male rats differ to some extent. Wake time was increased with age within 24 h [92], although Kostin et al. specified that this effect is observed in quiet wake and not in active wake [51]. NREMS remained unchanged with age during 24-h recordings [92, 101], however was reported to decrease during the light phase and increase during the dark phase in another study [51]. REMS time was decreased with age across 24 h in the early study [92]; however, recent studies observed a decrease in REMS only during the light phase [51, 101]. Despite the maintenance of NREMS, Shiromani and colleagues observed a decrease in delta power across 24 h [101], whereas similar to findings for NREMS, Rosenberg et al. [92] failed to find age-related differences in any frequency band. Similar to humans and mice, in Fisher 344 disturbed sleep-wake regulation with age was reflected by a more fragmented sleep with wake and sleep intrusions [51, 71, 92]. When sleep macrostructure across the lifespan was analyzed in females, the main difference to males was that aged Fisher 344 females had an increment in REMS during the dark phase [51]. Interactions with external variables were reported for aged F344 rats. For instance, exercise had positive consequences in the sleep EEG from old F344 rats [7]. However, no differences were observed when F344 rats were fed a hypocaloric diet [95].

Recordings of hippocampal ripple activity from the CA1 using tetrodes in male Fisher 344 rats demonstrated that the mean frequency of the ripples (90–240 Hz) was decreased by age, but not ripple density or duration [116]. Aged Fisher 344 rats reveal reduced extracellular levels of orexin (hypocretin, measured in their cerebrospinal fluid) [22]. In vivo microdialyses measured lower sensitivity of the adenosine A_1_ receptor in the basal forebrain of old Fisher 344 rats [71] reducing the somnogenic activity of adenosine [e.g., 56].

Male Sprague-Dawley rats showed no age-related differences in wake time, TST, NREMS, REMS, and number or duration of sleep and wake bouts or in the EEG spectral power of delta (0.5–4 Hz), SWA (2–8 Hz), or theta (6–9 Hz) [101, 118]. These results manifest the strain differences that can be found in the sleep EEG data in aged rats, even using the same experimental conditions.

Two studies investigated effects of aging in male Wistar rats; however, they used different analyses and comparison is difficult [17, 110]. Clément and colleagues found across 24 h more sleep fragmentation with age evidenced by a greater number yet shorter REMS episodes, and during the dark phase shorter wake episodes, more wake, NREMS, and REMS episodes [17]. In contrast, Van Gool and Mirmiran [110] found age-related changes during the light phase: more wake and less REMS. Delta (0.5–4 Hz) absolute power was found to be decreased only in a more recent study [17] while no changes were observed with age in theta power (4–11 Hz) [17, 110]. As observed in mice and in Fisher 344 rats, levels of orexin [97] and adenosine A_1_ receptor in the cortex are reduced in aged male Wistar rats as measured by histochemistry and analysis of their gene expression, respectively [15].

Similar to aging humans and mice, the three rat strains discussed above revealed a reduced compensatory increase in SWS or SWA after experimental sleep deprivation [17, 51, 101] indicating an altered homeostatic process of sleep with age.

Satinoff et al. [96] studied old Long Evans rats from both sexes and found an alteration in the peak neuronal discharge from the SCN, altering the circadian regulation and promoting sleep-wake instability. When studying only old female Long Evans rats, the age-related differences in sleep EEG parameters were observed with a decreased amplitude of the circadian rhythm of body temperature [57]. We are not aware of any further studies specifically targeting sleep in aging Long Evans rats.

## Conclusion

Effects of aging on brain electric activity in sleep are more consistent in humans than in rodents, revealing a need for more systematic studies on aging in rodents. Although sleep fragmentation is seen by humans and rodents alike, rodents do not reveal the distinguished age-related change in NREMS during the light (inactive) phase. Mice rather reveal increased sleep during the dark (active) phase denoting an increased homeostatic sleep drive, whereas aged humans express a reduced homeostatic sleep need. Yet in humans, systematic investigations on the interaction between homeostatic effects on both napping and nocturnal sleep and circadian process are required. Across species, a deficit in research on female subjects also prevails, a challenge to be overcome. Given the relevance of sleep for basic physiological functions, putative impacts on or interactions with sleep regulation require more intense research. Future research should build on findings underscoring not only system level but also recently disclosed molecular interactions between circadian and homeostatic processes. Translational research must hereby consider essential species-specific differences in regulatory mechanisms.
